# Supplementary material for: Roquin Modulates Cardiac Post-Infarct Remodeling via microRNA Stability Control
Source: Cells. 2025 Nov 7;14(22):1748. doi: 10.3390/cells14221748 (PMC12651655; doi:10.3390/cells14221748)
Supplement: Supplementary file 1 [file cells-14-01748-s001.zip › cells-3949127-supplementary.pdf]

mRNA-Primer	*Spezies*	*Annealing-Temperatur*	*Sequenz (5‘-3‘)*
B2M	*Human*	*59 °C*	*TGTCTTTCAGCAAGGACTGGT (for.)*<br>*ACATGTCTCGATCCCACTTAAC (rev.)*
B2M	*Ratte*	*65 °C*	*GCCGTCGTGCTTGCCATTC (for.)*<br>*CTGAGGTGGGTGGAACTGAGAC (rev.)*
HMGA2	*Human*	*60 °C*	*GCCCTCTCCTAAGAGACCCA (for.)*<br>*TTCGGCAGACTCTTGTGAGG (rev.)*
RC3H1	*Human*	*59 °C*	*GCTTACGAATTGCACCGGAC (for.)*<br>*TCCCAAGTAGGAGGAGGAGC (rev.)*
RC3H1	*Ratte*	*59 °C*	*GATCCCTGAGCAACAACCCA (for.)*<br>*TTGGGCGACTCAGAACACTC (rev.)*
RC3H2	*Human*	*58 °C*	*TGAACTTCAGCAGGCCAAGA (for.)*<br>*AGCATGAAGCCGAAAGGTTC (rev.)*
RC3H2	*Ratte*	*60 °C*	*CTGTCCAGCAGCACCAAAAG (for.)*<br>*TAATAGACACTGGGCGTGGC (rev.)*
ZBTB20	*Human*	*60 °C*	*GACACACACAGGAGTGAGGG (for.)*<br>*GTAGGACTTCTCTCCCCGGT (rev.)*
ZBTB20	*Ratte*	*60 °C*	*CTCCTCTATCGGCACCGAAC (for.)*<br>*GGAGAGGAGGCACCTGTTTC (rev.)*

microRNA-Primer	*Annealing-Temperatur*	*Sequenz (5‘-3‘)*
miR-23b-5p	*64 °C*	*GGGUUCCUGGCAUGCUGAUUU*
SNORD48	*64 °C*	*MystiCq Controlprimer Sigma (not specified)*

Suppl. Table 1: Information on PCR Primers

Above we provide the names of the primer pairs along with their annealing temperatures and sequences.
